# Unique clinical features and long term follow up of survivors of sudden cardiac death in an Asian multicenter study

**DOI:** 10.1038/s41598-021-95975-8

**Published:** 2021-09-14

**Authors:** Pang-Shuo Huang, Jen-Fang Cheng, Wen-Chin Ko, Shu-Hsuan Chang, Tin-Tse Lin, Jien-Jiun Chen, Fu-Chun Chiu, Lian-Yu Lin, Ling-Ping Lai, Jiunn-Lee Lin, Chia-Ti Tsai

**Affiliations:** 1 Division of Cardiology, Department of Internal Medicine, National Taiwan University Hospital Yun-Lin Branch, Yun-Lin County, Taiwan, ROC; 2 Division of Cardiology, Department of Internal Medicine, Ministry of Health and Welfare Pingtung Hospital, Pintung County, Taiwan, ROC; 3 Division of Cardiology, Department of Internal Medicine, Cathay General Hospital, Taipei, Taiwan, ROC; 4 Division of Cardiology, Department of Internal Medicine, Mennonite Christian Hospital, Hualien, Taiwan, ROC; 5 Division of Cardiology, Department of Internal Medicine, National Taiwan University Hospital Hsin-Chu Branch, Hsinchu City, Taiwan, ROC; 6 Division of Cardiology, Department of Internal Medicine, National Taiwan University Hospital , Taipei City, 100 Taiwan, ROC; 7 Cardiovascular Center, National Taiwan University Hospital, Taipei, Taiwan; 8 Division of Cardiovascular Medicine, Department of Internal Medicine, Taipei Medical University Shuang Ho Hospital, New Taipei City, Taiwan

**Keywords:** Cardiology, Cardiovascular diseases

## Abstract

There has been no long-term clinical follow-up data of survivors or victims of sudden cardiac death (SCD). The Taiwan multi-center sudden arrhythmia death syndrome follow-up and clinical study (TFS-SADS) is a collaborative multi-center study with median follow-up time 43 months. In this cohort, the clinical characteristics of these SADS patients were compared with those with ischemic heart disease (IHD). In this SCD cohort, around half (42%) were patients with IHD, which was different from Caucasian SCD cohorts. Among those with normal heart, most had Brugada syndrome (BrS). Compared to those with SADS, patients with IHD were older, more males and more comorbidities, more arrhythmic death, and lower left ventricular ejection fraction. In the long-term follow-up, patients with SADS had a better survival than those with IHD (p < 0.001). In the Cox regression analysis to identify the independent predictors of mortality, older age, lower LVEF, prior myocardial infarction and history of out-of-hospital cardiac arrest were associated with higher mortality and beta blocker use and idiopathic ventricular fibrillation or tachycardia (IVF/IVT) with a better survival during follow-up. History of prior MI was associated with more arrhythmic death. Several distinct features of SCD were found in the Asia–Pacific region, such as higher proportion of SADS, poorer prognosis of LQTS and better prognosis of IVF/IVT. Patients with SADS had a better survival than those with IHD. For those with SADS, patients with channelopathy had a better survival than those with cardiomyopathy.

## Introduction

Sudden cardiac death (SCD) describes an unexpected death from a cardiac cause within a short time period, generally less than 1 h from the onset of symptoms. SCD is one of the most common causes of death and a major and disastrous complication of cardiovascular diseases. SCD is often attributed to ventricular arrhythmia^1–4^. Several diseases linked to SCD have been reported^5^. In older populations, ischemic heart disease (IHD) which includes coronary artery disease (CAD) (chronic coronary artery obstruction) and myocardial infarction (MI) (acute coronary artery obstruction) account for the most majority of SCD^5,6^. However, in younger populations, SCD is usually caused by genetic diseases such as inherited arrhythmogenic cardiomyopathies including hypertrophic cardiomyopathy (HCMP), dilated cardiomyopathy (DCMP) and arrhythmogenic right ventricular cardiomyopathy (ARVC), and inherited cardiac ion channel diseases including Brugada syndrome (BrS), congenital long QT syndrome (LQTS), short QT syndrome (SQTS), catecholaminergic polymorphic ventricular tachycardia (CPVT) and idiopathic ventricular fibrillation or tachycardia (IVF/IVT). These inherited cardiac diseases predisposing to SCD are collectively known as sudden arrhythmia death syndromes (SADS)^1,7–10^.

Understanding the epidemiology and clinical characteristics of SCD and SADS allows the implementation of comprehensive and appropriate strategies for the prevention and treatment of SCD and SADS. The information of the long-term follow-up and outcomes of SCD and SADS victims is largely unknown, especially in the Asia–Pacific region. Whether the clinical characteristics of SCD due to IHD and those due to SADS are different is also unknown. The Taiwan SADS follow-up and clinical study (TFS-SADS) is a multi-center study that aims to collect comprehensive clinical data of SCD and SADS in Taiwan, which include disease entity, patient characteristics, long-term follow-up information and predictors of survival. In this study, we first described the data from the TFS-SADS, which provided the opportunity to look at and compare the clinical characteristics, long-term follow-up and outcome data of patients with different causes of SADS and SCD in the Asia–Pacific region.

## Methods

This was a multi-center retrospective cohort study monitoring patients of SCD and SADS in Taiwan. Victims or survivors of aborted SCD or SADS were enrolled in TFS-SADS from Jan. 2005 to Dec. 2016^11^ with median follow-up time 43 months. The specific objectives of this TFS-SADS were to determine factors associated with SCD and to study the clinical characteristics and predictors of outcomes of SCD and compare them between groups of different causes of SCD (inherited SADS or IHD) in Taiwan. At the end of this study, a total of 730 patients were enrolled and all patients were followed by their individual physicians in cardiology out-patient clinics. The demographic data, baseline characteristics, primary cardiac diagnosis, use of antiarrhythmic drugs and other cardiovascular medications, report of coronary angiography, results of electrophysiological study, echocardiographic data including left ventricular ejection fraction (LVEF) and clinical outcomes were collected. The study was approved by the Institutional Review Board of the National Taiwan University Hospital (No. 201705122RINC and No. 202107079RINC), informed consent is waived by IRB. The majority of the patients (627/730, 85.9%) underwent implantable cardioverter defibrillator (ICD) implantation, and thus this patient population represented a high risk SADS or SCD population.

The diagnoses of BrS, LQTS, SQTS and CPVT were according to the continuously updated guidelines from American Heart Association/American College of Cardiology (AHA/ACC) and European Society of Cardiology (ESC)^1,12,13^. The diagnosis of HCMP, ARVC or DCMP was according to the criteria of cardiac imaging studies including echocardiography, cardiac computed tomography (CT) or magnetic resonance imaging (MRI)^14–17^. The presence of coronary artery disease was documented by coronary angiography with the presence of at least one coronary stenosis more than 70% of the luminal diameter. The primary outcome was all-cause death. The secondary outcome was cardiovascular death which included arrhythmic and heart failure death. Arrhythmic death was defined as sudden death with witnessed or ICD-interrogated ventricular arrhythmia^1^. Heart failure death was defined as cardiovascular death other than arrhythmic death, which was usually associated cardiac pumping or circulatory failure.

### Statistical analysis

Continuous data were expressed as mean ± standard deviation (SD). The comparison was conducted by independent two-sample Student’s t-test for the continuous variables, and the chi-square test for the categorical variables. The cumulative mortalities or event rates were shown as survival curves in different groups by Kaplan–Meier method. The survival curves were compared using the log-rank test. The multivariable Cox proportional-hazards regression analysis was used to identify the predictors of survival, and hazard ratios (HRs) and their 95% confidence intervals (CIs) were calculated. The factors included in the multivariable model were those factors significant in the univariable analyses included age, gender, LVEF > 35%, Primary diagnosis (included prior MI, history of SCD), amiodarone or beta blocker use.

Survival analyses were also stratified by the presence or absence of IHD. In those SADS, the analysis was further stratified by the presence or absence of cardiomyopathy. All tests were two sided, and a significance value of p < 0.05 was used. All statistical analyses were carried out using the SPSS 23.0 (SPSS Inc. USA).

### Ethics, consent and permissions

The study was approved by the Institutional Review Board of the National Taiwan University Hospital (No. 201705122RINC and No. 202107079RINC), informed consent is waived by IRB. This is a retrospective cohort and the delinked medical data were collected for analyses without consents from the participants.

## Results

### Baseline characteristics

Table 1 shows the primary diagnoses of all the enrolled patients. Among them, 42% (305/730) were patients with ischemic heart disease (IHD). This result was similar to our previous report that in the Asia–Pacific region, the proportion of SCD due to IHD was lower compared to that in the Caucasian populations in which nearly 80% of the SCD patients were due to IHD^11,18,19^.

**Table 1:** Primary cardiac diagnosis of patients with sudden cardiac death.

Primary cardiac diagnosis	Cohort n = 730 (%)
**CAD**	305 (42%)
Prior myocardial infraction	176 (24%)
CABG	81 (11%)
3-V-D	160 (22%)
**Nonischemic cardiomyopathy**	201 (27%)
Dilated cardiomyopathy	93 (13%)
Hypertrophic cardiomyopathy	80 (11%)
Alcoholic cardiomyopathy	3 (0.4%)
Right ventricular dysplasia	25 (3%)
**Other structural heart disease**	68 (9%)
Valvular heart disease	45 (6%)
Other*	23 (3%)
**Normal structural heart disease**	156 (21%)
Brugada syndrome	61 (8%)
Idiopathic VF	35 (5%)
Idiopathic VT	26 (4%)
Long QT syndrome	25 (4%)
Short QT syndrome	6 (1%)
Catecholaminergic polymorphic ventricular tachycardia	3 (0.4%)

*CAD* coronary artery disease; *CABG* coronary artery bypass grafting; *3-V-D* three vessel disease; *VF* ventricular fibrillation; *VT* ventricular tachycardia.

*Other: TGA (Transposition of the great arteries), TOF (Tetralogy of Fallot), Amyloidosis, LV aneurysm.

For those patients with SCD not caused by IHD, the majority were caused by genetic diseases such as inherited arrhythmogenic cardiomyopathies or cardiac ion channel diseases (channelopathy). For comparison with those caused by IHD, they were collectively called as SADS in the present study. In those patients with non-IHD or SADS, around one third had normal structure heart and the other two thirds had cardiomyopathy (HCMP, ARVC and DCMP). Among all the SADS patients with normal structure heart, most of the patients had BrS, compatible with previous reports that BrS was the most common cause of SCD in patients with SCD and normal structure heart in the Asia–Pacific region^20,21^.

The comparison of clinical characteristics between those with IHD and SADS is shown in Table 2. Patients with IHD were older (66.1 vs. 51.3 year-old, p < 0.001) and had a male predominance (80% vs 68%, p = 0.001) and more comorbidities such as diabetes (34% vs 13%, p < 0.001), hyperlipidemia (40% vs 14%, p < 0.001), chronic kidney disease (38% vs 21%, p < 0.001), more death due to arrhythmia (3% vs 1%, p = 0.010) and heart failure (7% vs 4%, p = 0.043), higher mortality (22% vs 7%, p < 0.001), more use of amiodarone (58% vs 30%, p < 0.001) and poorer LVEF (43% vs 55%, p < 0.001). In patients with SADS, the comparison of clinical characteristics between patients with cardiomyopathy and those with normal structure heart patients is shown in Table 3. Patients with cardiomyopathy were older and more of them had chronic kidney disease and death due to heart failure, and had a poor left heart ejection fraction. So, those SADS patients with a normal heart were younger, and could be considered as the population of SCD in the young.

**Table 2:** Comparisons of clinical characteristics of patients in the presence and absence of ischemic heart disease during follow-up.

Variables	IHD (n = 305)	SADS (n = 425)	P value
Age (years)	66.1 ± 12.8	51.3 ± 18.8	< 0.001
Male sex	243 (80%)	289 (68%)	0.001
Left ventricular ejection fraction (%)	43.0 ± 17.5	55.3 ± 18.3	< 0.001
Hypertension	63 (21%)	74 (17%)	0.290
Diabetes	66 (34%)	43 (13%)	< 0.001
Hyperlipidemia	77 (40%)	45 (14%)	< 0.001
Chronic kidney disease	116 (38%)	89 (21%)	< 0.001
Amiodarone use	176 (58%)	125 (30%)	< 0.001
Beta blocker use	188 (62%)	239 (57%)	0.146
**Death**	66 (22%)	28 (7%)	< 0.001
Arrhythmic	10 (3%)	3 (1%)	0.010
Heart failure	20 (7%)	15 (4%)	0.043
Non-cardiac	34 (11%)	12 (3%)	< 0.001

*IHD* Ischemic heart disease, *SADS* Sudden arrhythmia death syndromes.

**Table 3:** Comparisons of clinical characteristics of patients with cardiomyopathy and normal structure heart patients in the SADS group during follow-up.

Variables	Cardiomyopathy	Normal structure heart	*P* value
	(n = 269)	(n = 156)	
Age (years)	56.6 ± 17.5	45.4 ± 16.8	< 0.001
Gender (male%)	188 (70%)	109 (70%)	0.942
Left ventricular ejection fraction (%)	50.2 ± 20.4	63.1 ± 11.5	< 0.001
Hypertension	56 (21%)	27 (17%)	0.285
Diabetes	43 (16%)	16 (10%)	0.121
Hyperlipidemia	46 (17%)	19 (12%)	0.167
Chronic kidney disease	70 (26%)	22 (14%)	0.003
Amiodarone use	100 (37%)	22 (14%)	< 0.001
Beta blocker use	178 (66%)	70 (45%)	< 0.001
**Death**	19 (7%)	3 (2%)	0.019
Arrhythmic	3 (1%)	0 (0%)	0.246
Heart failure	13 (5%)	0 (0%)	0.004
Non-cardiac	3 (1%)	3 (2%)	0.618

*SADS* sudden arrhythmic death syndrome.

Among these SCD victims, 85.9% (n = 627) of the patients received ICD implantation, but not all of the patients, because the majority of indication of ICD implantation was a secondary prevention because of the national health insurance reimbursement policy.

### Long-term follow up of patients with SCD

This is a long-term follow-up study of SADS population. We have the opportunity to compare the outcome of patients with different etiologies of SCD, e.g., comparing the outcome of patients with IHD and that of those with hereditary cardiac diseases or SADS.

**Figure 1:**
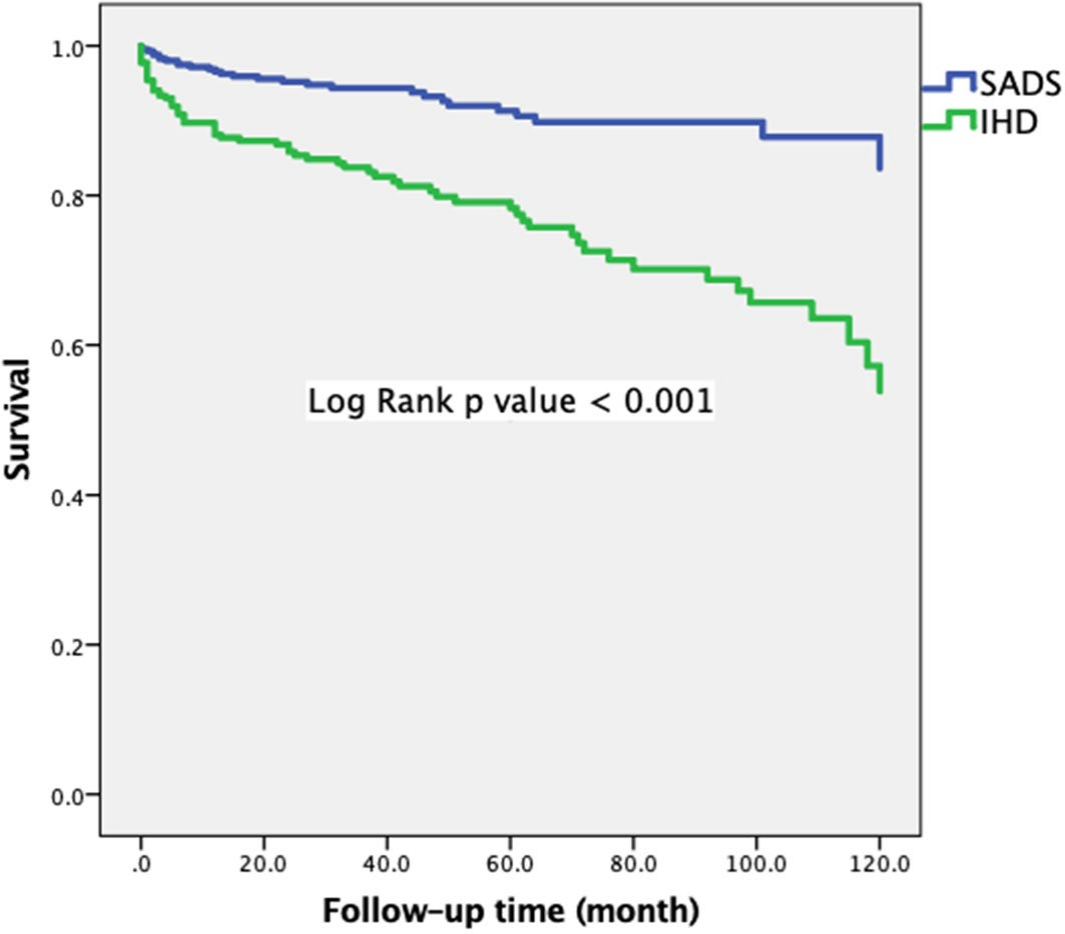
Kaplan–Meier curve according ischemic heart disease.

We first compared the long-term survivals of patients with non IHD or SADS and those with IHD and the result is shown in Fig. 1. In general, those with SADS had a better survival than those with IHD (p < 0.001).

**Figure 2:**
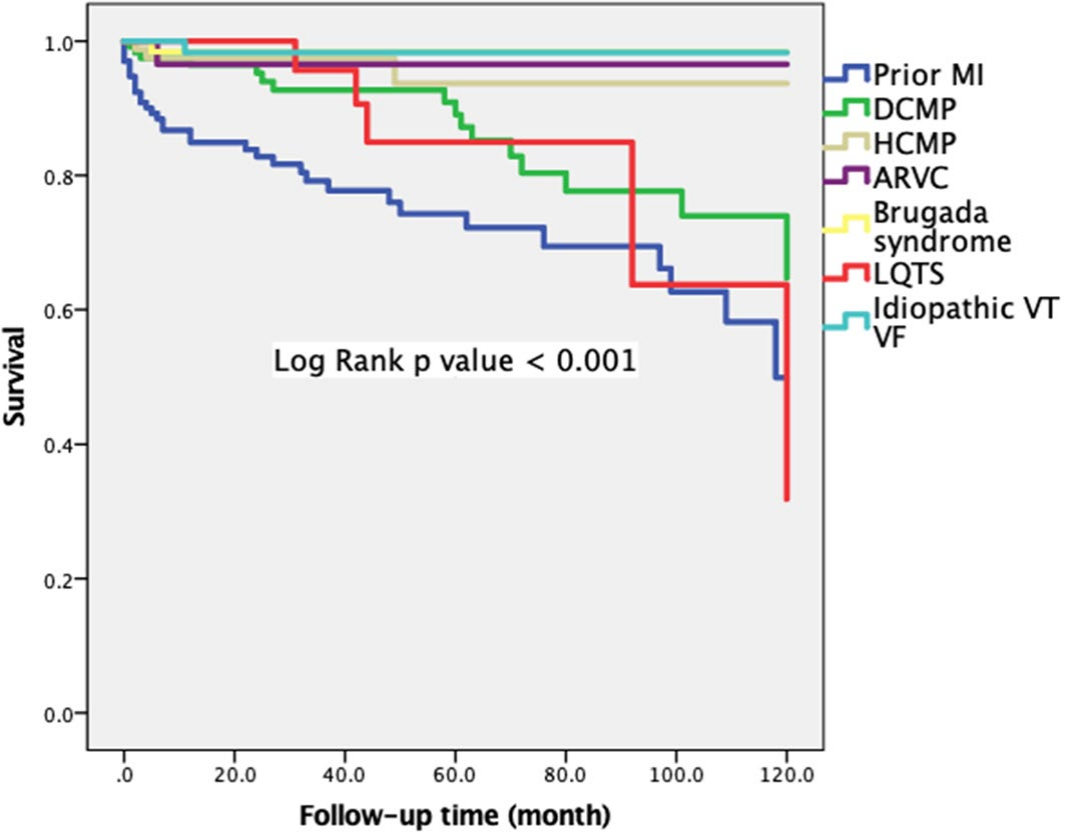
Kaplan–Meier curve according etiology.

We then compared the survivals of all the patients according to the primary diagnosis and the result is shown in Fig. 2. Interestingly, the survivals of patients with different etiologies of SCD were significantly different from one another (p < 0.001). Between different etiologies of SCD, patients with prior MI, DCMP and LQTS had the worst outcome (p < 0.001 for those with MI or DCMP or LQTS vs. others). The patients with LQTS had a poorer survival as those with prior MI or DCMP, although they had a normal heart.

Among those patients with hereditary cardiac diseases or SADS (channelopathy and cardiomyopathy), we found patients with IVF/IVT had the most favorable outcome (Supplementary Fig. S1) (p = 0.007). Again, patients with DCMP and LQTS had the least favorable outcome (Supplementary Fig. S2) (p = 0.015). We further dichotomized those SADS patients into those with a normal heart (channelopathy) and those with cardiomyopathy (HCMP, ARVC and DCMP) and compared the long-term survivals of these two groups (Supplementary Fig. 3). We found a significant difference of survival between these two groups. SADS patients with cardiomyopathy had a poorer survival than those with normal heart (p = 0.001).

### Independent predictors of long-term survivals for patients with SCD

We first used multivariable Cox regression analysis to investigate the independent predictors of mortality for patients with SCD. For the entire study population, we found older age, lower LVEF, history of prior MI and history of out-of-hospital cardiac arrest (OHCA) (borderline statistically significant) were associated with higher mortality during follow-up (HR 2.565 [95% CI 1.679–3.920], p < 0.001; HR 0.592 [95% CI 0.386–0.906], p = 0.016; HR 1.827 [95% CI 1.163–2.870], p = 0.009; HR 1.523 [95% CI 0.996–2.327], p = 0.052). Patients with IVF/IVT had a better survival (Hazard ratio (HR) 0.137 [95% CI 0.019–1.004], p = 0.050).

**Figure 3:**
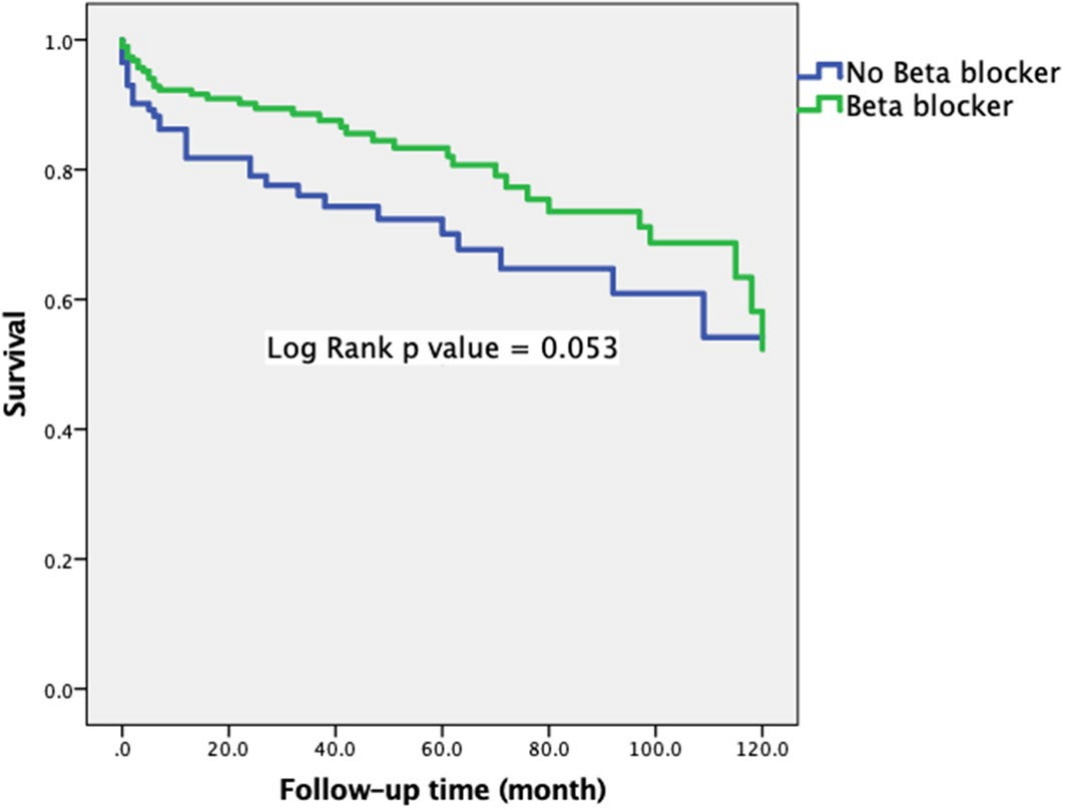
Kaplan–Meier curve within ischemic heart disease according beta blocker use.

**Figure 4:**
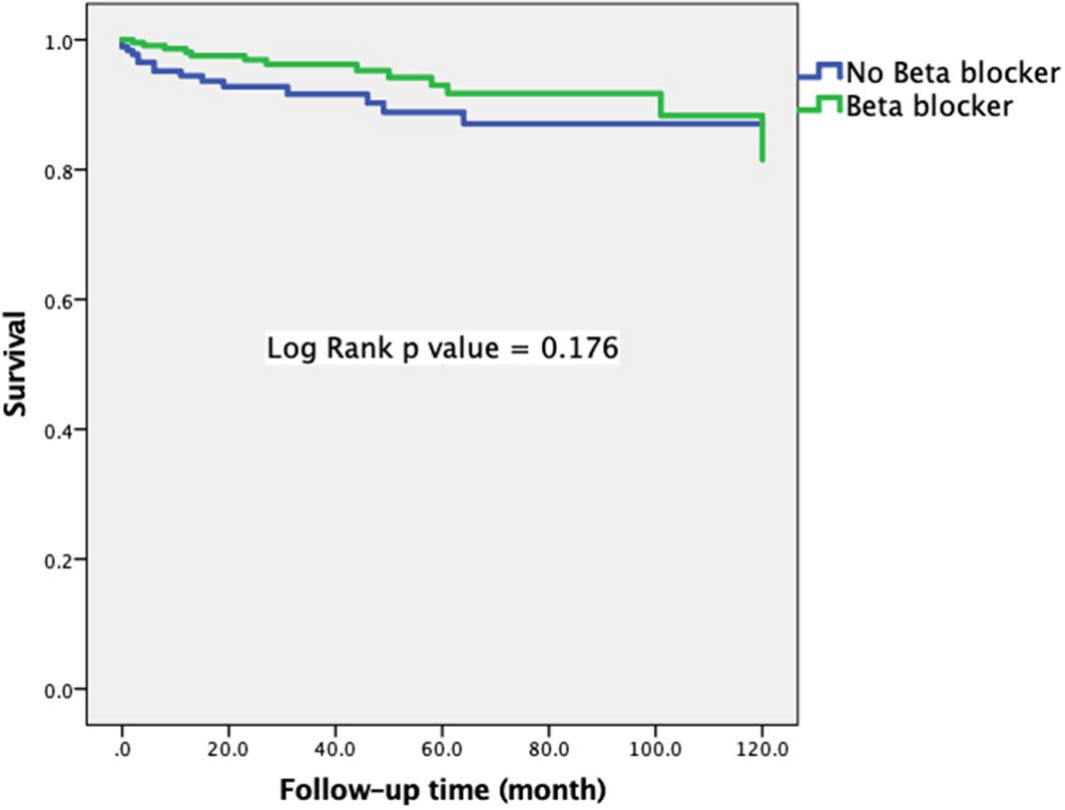
Kaplan–Meier curve within SADS according beta blocker use.

Interestingly, we also found beta blocker use was also associated with a better survival (Hazard ratio (HR) 0.614 [95% CI 0.407–0.926], p = 0.020). To identify specific populations which might benefit from beta blocker use. We did analyses in subgroups. As expected, for those patients with IHD, use of beta blocker was associated with a better outcome (HR 0.621, p = 0.056) (Fig. 3), but only borderline statistically significant. However, use of beta blocker was not associated with a better outcome for those SADS patients (HR 0.602, p = 0.180) (Fig. 4).

We also used multivariable Cox regression analysis to investigate the independent predictors of mortality for patients with hereditary cardiac diseases or SADS (channelopathy and cardiomyopathy). We found older age and lower LVEF were associated with higher mortality during follow-up (HR 3.472 [95% CI 1.566–7.695], p = 0.002 for age > 65 years, and HR 2.315 [95% CI 1.025–5.208], p = 0.043 for LVEF < 35%, respectively).

Because these patients were susceptible to ventricular arrhythmia, we also looked at the predictors of death due to ventricular arrhythmia. Among the total of 730 patients at the end of follow-up, 13 were died of arrhythmic death. We found history of prior MI was the only independent predictor of arrhythmic death (HR 3.778 [95% CI 1.270–11.243], p = 0.017) (Supplement Table S1). Age older than 65 years and LQTS were also associated with arrhythmic death with borderline significant p values (HR 3.046 [95% CI 0.996–9.316, p = 0.051 and HR 3.877 [95% CI 0.858–17.509], p = 0.078, respectively] (Supplement Table S1).

## Discussion

This is the first long-term follow-up study of SCD victims and also the first follow-up study of SADS victims in the Asia–Pacific region. So far the demographics, clinical features and long-term outcome of SADS or SCD have not been well characterized in the Asia–Pacific region. We identified several predictors of survival for patients with SCD or SADSs. These results may provide useful information in risk stratification.

Most of the previous SCD studies are cross-sectional and focused on postmortem examination^22–26^. Here we first described the clinical characteristics and follow-up information of survivors and victims of SADS or SCD in the Asia–Pacific region. The study on survivors or victims of SCD provides additional information regarding the future care of these patients, but not only the information about the distribution or percentage of various causes of SADS as revealed in previous cross-sectional studies^22–26^.

### Etiology of SCD in the Asia–Pacific region and comparison with those of Caucasian population

Several distinct clinical features have been found in the survivors and victims of SCD in the present study, compared to those of the Caucasian populations. In our Asian SCD population, 37% (156/425) of them are BrS or normal heart, the percentage of which is higher compared to that of Caucasian SCD populations^27,28^. LQTS or CPVT is less common. Previously we had shown that the percentage of IHD was lowered in our SCD population, compared to that of Caucasian populations^11,27,28^. In this expanded population with a longer follow-up, we still had a similar finding.

This phenomenon may reflect different incidences of SCD in patients with IHD in different ethnic populations. It has been reported that the incidence of coronary deaths occurring outside of the hospital or in emergency rooms was lower in western populations^29^. Furthermore, in a survey of sudden death of adults in Japan in 1996, sudden death due to IHD was more in Western countries than in Japan^30^. Therefore, it is possible that the lower percentage of IHD in the present SCD population may represent a lower incidence of SCD in patients with IHD in the Asia–Pacific region.

In the patients with normal heart, in addition to BrS, there was also a higher proportion of patients with IVF or IVT. We believe that some of them might have Brugada syndrome or CPVT since resting or even provocative ECG of these two syndromes might be normal and challenges exist for a definite diagnosis. In this scenario, genetic test may help. However, the rate of sodium channel mutation (*SCN5A*) mutation is low in Asian patients with BrS^31,32^ and the rate of background mutations in ryanodine receptor 2 gene (*RYR2*) or calsequestrin 2 gene (CASQ2) are substantially high^33^, which sometimes make the definite diagnosis of BrS and CPVT even more difficult. Finally, a similar result has been reported that the percentage of IVF or IVT was as high as up to 44.1% in a survey of unexplained sudden cardiac death in Korea^34^. Therefore, it is still possible that the percentage of IVF or IVT is higher among the causes of SCD in the Asian populations.

### Predictors of long-term survival for SADS patients

Our study also reveals several unique predictors of survival in the long-term follow-up of SADS or SCD victims, which has never been reported before. More care should be given and more frequent follow-up should be delivered to those with risk factors of mortality. The information of predictor of survival or mortality also provides the opportunity of early warning and explanation to the families and possibly a stronger persuasion of a genetic test for the at-risk family members.

As expected, for the entire SCD population, older age, history of MI and lower LVEF were associated with higher mortality. Use of beta blocker was associated with a better survival. In the subgroup analysis, use of beta blocker was associated with a better survival in those with IHD but not in those with non-IHD or SADS. However, it has been shown that use of beta blocker prolonged the survivals of patients with DCMP, HCMP or LQTS^35–39^. In our SADS population, patients with BrS and IVF accounted for a significant proportion of the population, who may not benefit from use of beta blocker. This may explain why use of beta blocker was not associated with a better survival in our SADS population.

Our study also first shows that patients with SADS had a better survival than those with IHD. And, in those with SADS, patients with a normal heart had a better survival than those with cardiomyopathy, in which those with DCMP had the worst outcome. The reason is straightforward, since patients with IHD or DCMP were generally older and had poorer LV function, and more comorbidities such as hypertension, diabetes or hyperlipidemia. Furthermore, even though a great progress in the technology of percutaneous coronary intervention, the mortality of patients with IHD, especially those with impaired left ventricular systolic function still keeps in a constantly high level^40^. And, for those with DCMP, in addition to susceptibility to SCD, they might also develop progressive deterioration of cardiac function, which might increase the incidence of both cardiac and non-cardiac deaths. Finally, the better survival of SADS patients with a normal heart indicates that SADS with a normal heart is a treatable disease if early prevention and treatment with ICD are given to those at-risk patients.

Our study also provides the opportunity to specifically look at and compare the survivals of patients with each different cause of SCD. Patients with history of MI, DCMP and LQTS had the worst outcome and patients with IVF or IVT had the best outcome. The long-term survival rate of LQTS was as low as those with DCMP or those with a prior MI, which was really out of expectation and has never been reported before. In the Caucasian registry, it has been reported that LQTS was a potentially lethal cardiac channelopathy with a 1% to 5% annual risk of LQTS-triggered syncope, aborted cardiac arrest, or sudden cardiac death^41^. Although the case number is not high in the present study, we first show that the annual rate of mortality was around 5% for the Asian LQTS patients, which reached the the upper limit of the reported SCD rate (1–5%). The percentage of beta blocker use was similar in our LQTS population (50%) compared to that reported in the Caucasian population (58%)^41^ and it is not likely that the high rate of mortality in our LQTS patients is due to low rate of beta blocker use.

LQTS is rare in the Asia–Pacific region. Therefore, most of the LQTS patients were recognized by the presentation of aborted SCD and therefore most of them are symptomatic and the treatment with ICD is based on a secondary prevention attempt. In the Caucasian LQTS registry^41^, symptomatic patients accounted for only 25% of the total study population. That may be the reason why the prognosis of LQTS may be poorer in the Asian LQTS registry since most recruited LQTS patients are symptomatic or have a history of aborted SCD.

Arrhythmia death despite ICD treatment represents a devastating medical condition in which although there is ICD shock treatment, the patients still die due to refractory heart failure, respiratory failure or failure to convert to sinus rhythm^42^. This population represents the non-ICD treatable and most dangerous population and needs more attention. Our study first identified the risk factors of arrhythmia death in the SCD survivors. We found patients with history of MI, older age and LQTS were probably more susceptible to arrhythmic death. Therefore, the follow-up and treatment should be more scrutinized in these patients, such as more frequent ICD interrogation to detect non-sustained ventricular arrhythmia, more aggressive medical treatment or higher dose of beta blocker, and avoid drugs that predispose to ventricular arrhythmia (class I anti-arrhythmic drugs)^1,13,18^. For those with prior MI and history of SCD or ventricular arrhythmia, early and more frequent stress test to detect subtle myocardial ischemia, institution of early coronary intervention, and more intensive statin treatment may be warranted^43–45^.

## Conclusions

Several distinct features of SCD were found in the Asia–Pacific region, such as higher proportion of SADS, poorer prognosis of LQTS and better prognosis of IVF/IVT. Patients with SADS had a better survival than those with IHD. For those with SADS, patients with channelopathy had a better survival than those with cardiomyopathy.

### Statement

All methods were carried out in accordance with relevant guidelines and regulations.

### Limitations

Our study has limitations. First, because of the high screen rate of echocardiography and ECG in our pediatric patients, SCD due to congenital heart disease and Wolf-Parkinson-White syndrome are absent in our SADS population. Second, the case numbers of some diseases, especially channelopathy such as LQTS, CPVT and SQTS are relatively low and a higher power of statistical analysis could only be obtained by joint analyses.

## Supplementary Information

Supplementary Figure 1.

Supplementary Figure 2.

Supplementary Figure 3.

Supplementary Tables.

## Abbreviations

ARVC: Arrhythmogenic right ventricular cardiomyopathy
BrS: Brugada syndrome
CAD: Coronary artery disease
CPVT: Catecholaminergic polymorphic ventricular tachycardia
CT: Computed tomography
DCMP: Dilated cardiomyopathy
HCMP: Hypertrophic cardiomyopathy
HR: Hazard ratio
ICD: Implantable cardioverter-defibrillator
IHD: Ischemic heart disease
IVF/VT: Idiopathic ventricular fibrillation or tachycardia
LVEF: Left ventricular ejection fraction
LQTS: Long QT syndrome
MI: Myocardial infarction
MRI: Magnetic resonance imaging
OHCA: Out of hospital cardiac arrest
SADS: Sudden arrhythmia death syndromes
SCD: Sudden cardiac death
SD: Standard deviation
SQTS: Short QT syndrome
TFS-SADS: Taiwan sudden arrhythmia death syndrome study
VT: Ventricular tachycardia
VF: Ventricular fibrillation
