# Supplementary figures and images for: Unique clinical features and long term follow up of survivors of sudden cardiac death in an Asian multicenter study
Source: Sci Rep. 2021 Sep 14;11:18250. doi: 10.1038/s41598-021-95975-8 (PMC8440502; doi:10.1038/s41598-021-95975-8)

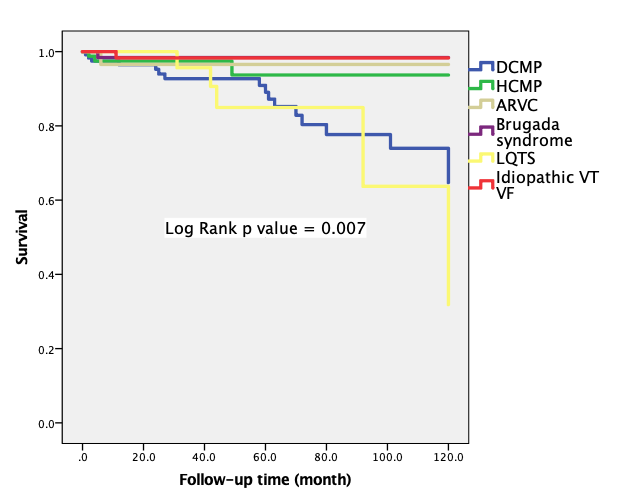

Supplement: Supplementary file 1 — Supplementary Figure 1. [file 41598_2021_95975_MOESM1_ESM.tiff]

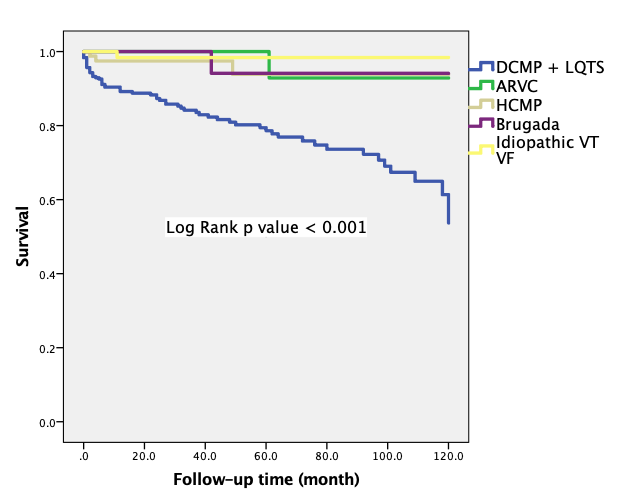

Supplement: Supplementary file 2 — Supplementary Figure 2. [file 41598_2021_95975_MOESM2_ESM.tiff]

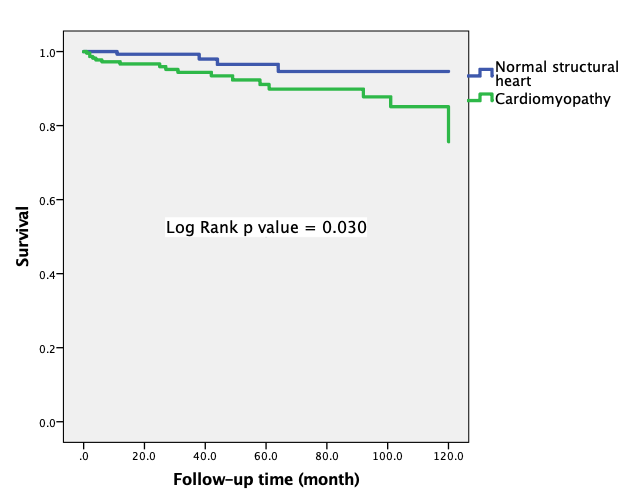

Supplement: Supplementary file 3 — Supplementary Figure 3. [file 41598_2021_95975_MOESM3_ESM.tiff]
