# Supplementary material for: Unique clinical features and long term follow up of survivors of sudden cardiac death in an Asian multicenter study
Source: Sci Rep. 2021 Sep 14;11:18250. doi: 10.1038/s41598-021-95975-8 (PMC8440502; doi:10.1038/s41598-021-95975-8)
Supplement: Supplementary file 4 — Supplementary Tables. [file 41598_2021_95975_MOESM4_ESM.docx]

**Unique clinical features and long term follow up of survivors of sudden cardiac death in an Asian multicenter study**

Running title: Sudden death and Long-Term Prognosis

Pang-Shuo Huanga, Jen-Fang Chengb, Wen-Chin Ko_c_, Shu-Hsuan Chang_d_, Tin-Tse Line, Jien-Jiun Chena, Fu-Chun Chiua, Lian-Yu Linf, Ling-Ping Laif, Jiunn-Lee Linf, Chia-Ti Tsaif

aDivision of Cardiology, Department of Internal Medicine, National Taiwan University College of Medicine and Hospital Yun-Lin Branch, Yun-Lin County, Taiwan

bDivision of Cardiology, Department of Internal Medicine, Ministry of Health and Welfare Pingtung Hospital, Pintung County, Taiwan

cDivision of Cardiology, Department of Internal Medicine, Cathay General Hospital, Taipei, Taiwan

dDivision of Cardiology, Department of Internal Medicine, Mennonite Christian Hospital, Hualien, Taiwan

eDivision of Cardiology, Department of Internal Medicine, National Taiwan University College of Medicine and Hospital Hsin-Chu Branch, Hsinchu City, Taiwan

fDivision of Cardiology, Department of Internal Medicine, National Taiwan University College of Medicine and Hospital, Taipei, Taiwan

Address for correspondence: Chia-Ti Tsai, MD, PhD, Division of Cardiology, Department of Internal Medicine, National Taiwan University College of Medicine and Hospital No. 7, Chung-Shan South Road, Taipei 100, Taiwan. Phone: 886-2-23123456. Fax: 886-2-82317099. E-mail: cttsai1999@gmail.com.

FUNDING SOURCES

The authors declare no conflict of interest.

Supplement Table 1. Mutivariable analysis of arrhythmia death.

| Variables |  | Hazard ratio (95% CI) |  | *p* value |
| --- | --- | --- | --- | --- |
| Age>65 |  | 3.046 (0.996-9.316) |  | 0.051 |
| Sex (Male) |  | 1.251 (0.344-4.547) |  | 0.734 |
| LVEF<35% |  | 2.217 (0.745-6.623) |  | 0.152 |
| Primary diagnosis |  |  |  |  |
| Prior MI |  | 3.778 (1.270-11.243) |  | 0.017 |
| DCMP |  | 0.038 (0-22.320) |  | 0.314 |
| HCMP |  | 1.347 (0.298-6.083) |  | 0.699 |
| ARVC |  | 0.046 (0-6033) |  | 0.609 |
| Brugada syndrome |  | 0.043 (0-211.997) |  | 0.469 |
| LQTS |  | 3.877 (0.858-17.509) |  | 0.078 |
| Idiopathic VT / VF |  | 0.042 (0-98.285) |  | 0.423 |
| History of SCD |  | 2.003 (0.673-5.961) |  | 0.212 |
| Medication |  |  |  |  |
| Amiodarone use |  | 2.998 (0.923-9.741) |  | 0.068 |
| Beta blocker use |  | 0.548 (0.184-1.631) |  | 0.28 |

LVEF, left ventricular ejection fraction; MI, myocardial infarction; DCMP, dilated cardiomyopathy; HCMP, hypertrophic cardiomyopathy; ARVC, arrhythmogenic right ventricular cardiomyopathy; CI, confidence interval; LQTS, long QT syndrome; VT, ventricular tachycardia; VF, Ventricular fibrillation; SCD, Sudden cardiac death.

Supplement Table 2. Mutivariable analysis of death in SADS group

| Variables |  | Hazard ratio (95% CI) |  | *p* value |
| --- | --- | --- | --- | --- |
| Age>65 |  | 3.472 (1.566-7.695) |  | 0.002 |
| Sex (Male) |  | 0.650 (0.300-1.409) |  | 0.275 |
| LVEF<35% |  | 2.315 (1.025-5.208) |  | 0.043 |
| Primary diagnosis |  |  |  |  |
| Prior MI |  | 3.191 (0.394-25.821) |  | 0.277 |
| History of SCD |  | 2.037 (0.946-4.387) |  | 0.069 |
| Medication |  |  |  |  |
| Amiodarone use |  | 1.267 (0.573-2.799) |  | 0.559 |
| Beta blocker use |  | 0.558 (0.259-1.202) |  | 0.136 |

LVEF, left ventricular ejection fraction; MI, myocardial infarction; VT, ventricular tachycardia; VF, Ventricular fibrillation; SCD, Sudden cardiac death.
